# P-1514. ID vs ED: Rabies Vaccine Cost, Time, and Completion Comparison

**DOI:** 10.1093/ofid/ofaf695.1698

**Published:** 2026-01-11

**Authors:** Timothy Counce, Ryan Manthey, Hassan El-Chebib, Ashley Howard

**Affiliations:** Connecticut Childrens Medical Center, Hartford, CT; Connecticut Childrens, Hartford, Connecticut; University of Connecticut, Hartford, Connecticut; University of Connecticut, Hartford, Connecticut

## Abstract

**Background:**

Rabies is a 100% fatal virus transmitted mainly by animal saliva through breaks in the skin or mucus membranes. Children are especially at risk given the inherent higher level of injuries necessitating additional medical/surgical care. Post exposure prophylaxis is recommended with a 4-dose vaccine series and immunoglobulin. Most vaccines are administered in the Emergency Department (ED), with variable costs and adherence to schedule. The CDC Yellow book estimates per-vaccine costs of around $400. This project aimed to examine vaccine administration costs, encounter time, and completion rates between patients seen at Connecticut (CT) Children’s outpatient Infectious Disease (ID) clinic vs (ED).

**Figure:**
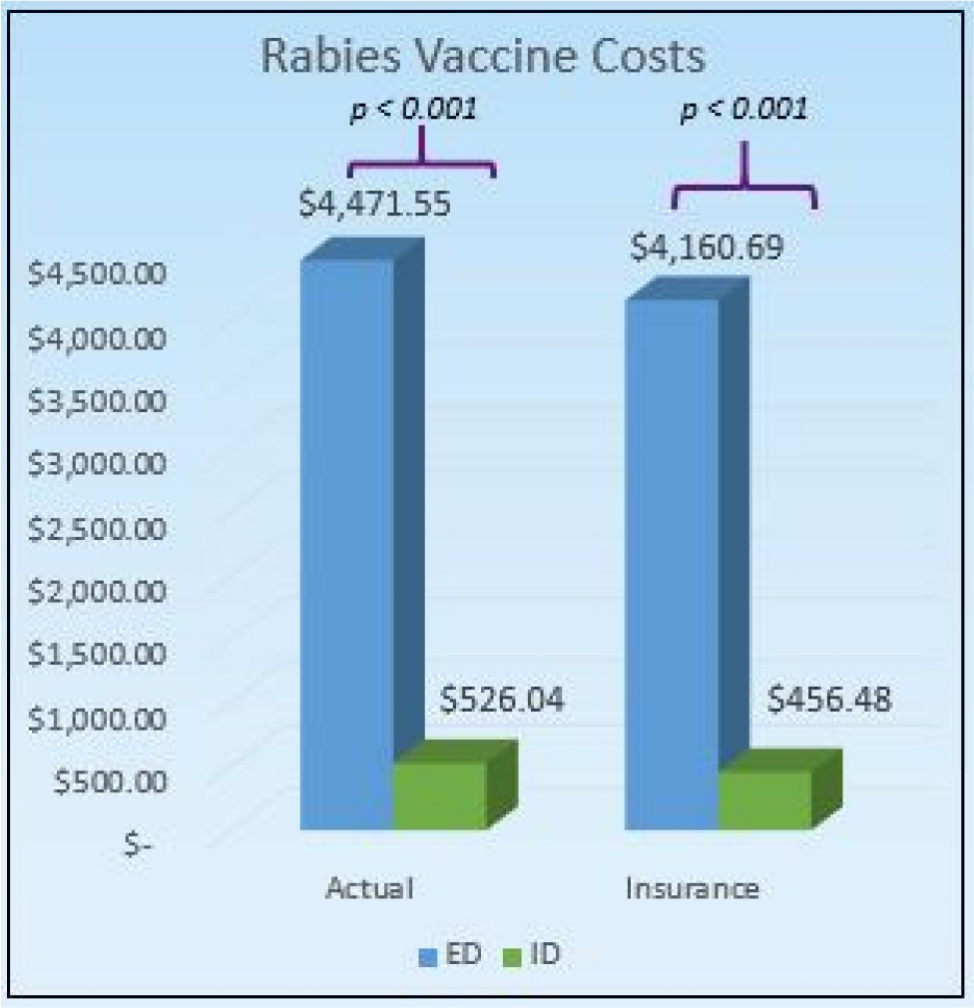
Figure 1

**Figure:**
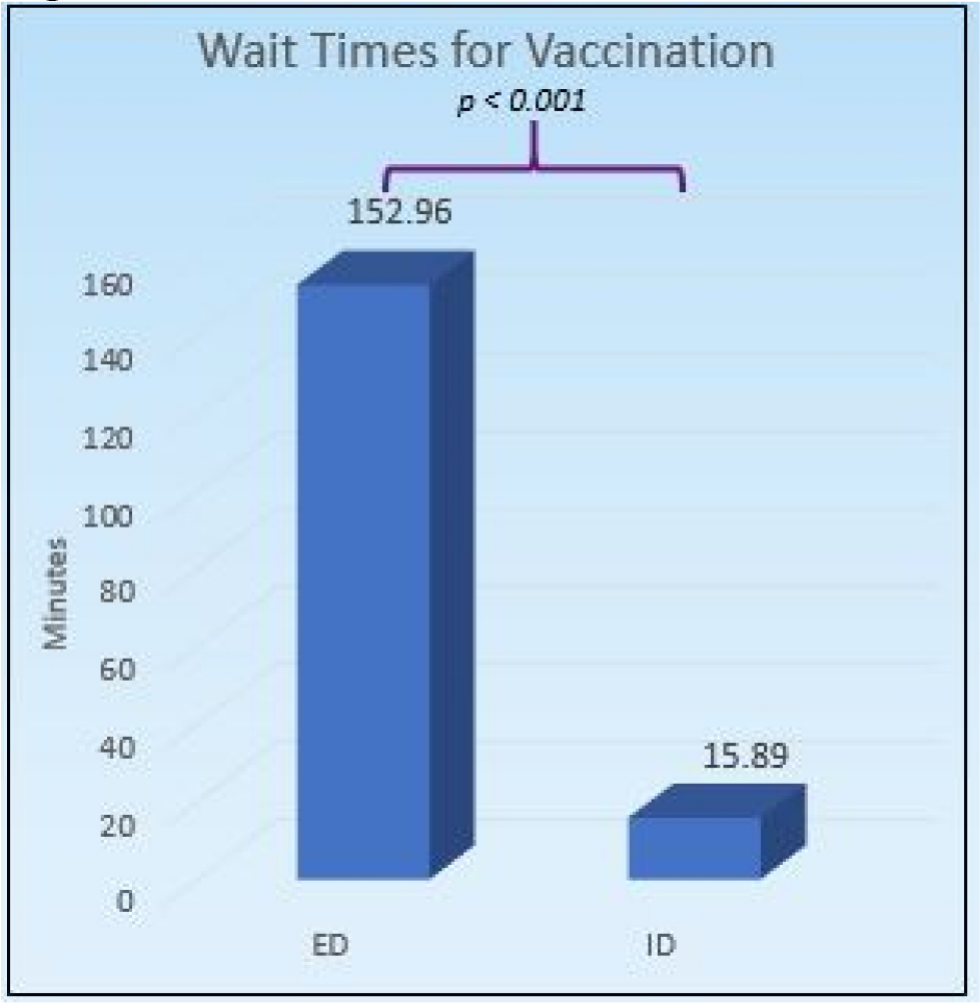
Figure 2

**Methods:**

Retrospective study of any person regardless of age who presented to CT Children’s ED between 11/01/2017 to 12/31/2022. Patients were captured using ICD10 codes. 350 persons met inclusion criteria. 256 patients completed the entire rabies vaccine series. 340 patients had cost data for the ED and 63 for ID visits. Pandemic start was 03/11/2020. Data were analyzed via REDCap and SPSS. Wilcoxon rank-sum and Wilcoxon signed-rank tests utilized where appropriate. All testing were two-sided, and statistical significance was defined as a p-value < 0.05.

**Figure:**
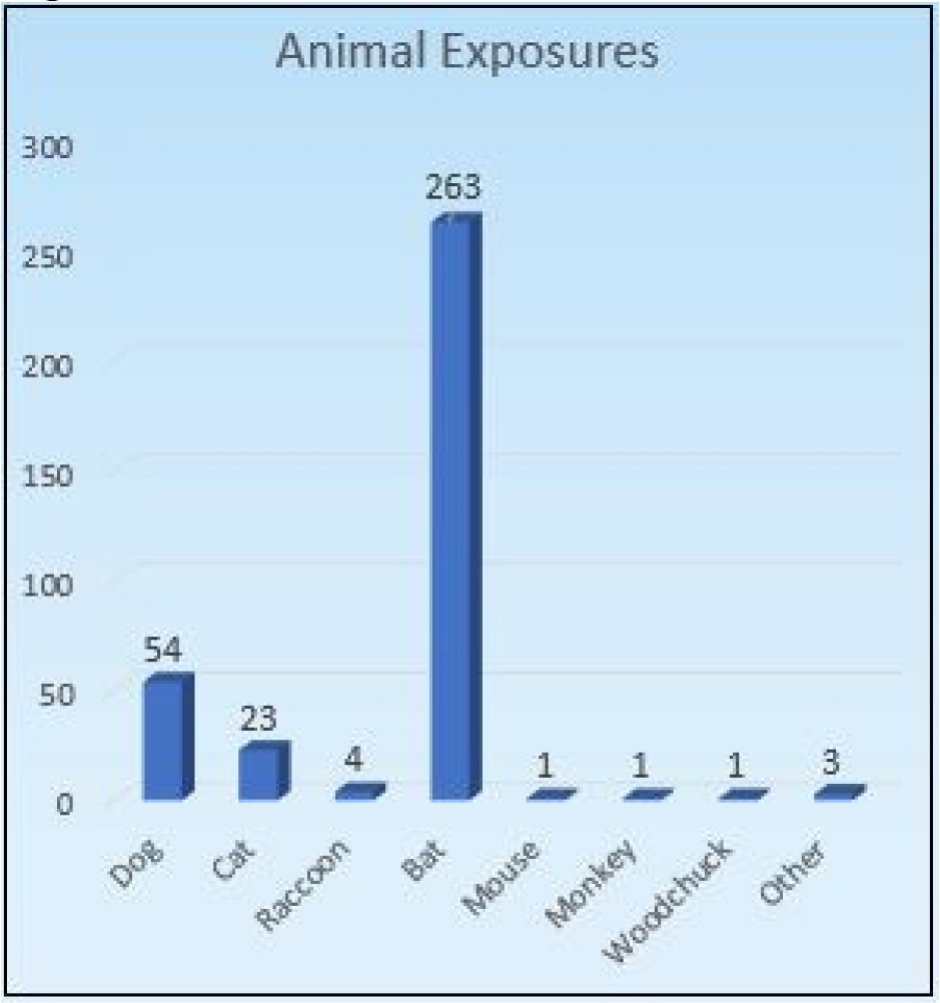
Figure 3

**Results:**

The mean costs billed for ED and ID visits were $4,471.55 and $526.04, respectively (*p* < 0.001). Insurance reimbursed a mean of $4,160.69 and $456.48 for ED and ID visits, respectively (*p* < 0.001). Bats were the most common source of exposure at 236. Pre-pandemic completion rates were 61%, and post-pandemic completion rates were 83.3% (*p* < 0.001). Mean wait times for ED visits were 152.96 minutes and for ID visits were 15.89 minutes (*p* < 0.001) There was no significant difference in care cost between those above and below 18 years of age.

**Conclusion:**

The ID outpatient clinic is an economical alternative to ED visits for rabies vaccine completion and an estimated savings of $11,836.53. Vaccination series completion rates, regardless of setting, were higher after the COVID-19 pandemic compared to those that were exposed to rabies before the pandemic. In addition, despite the inherent higher severity of animal-related injuries in children, there are was no significant cost difference for medical care between children and adults.

**Disclosures:**

All Authors: No reported disclosures

